# BCR–ABL1-induced downregulation of WASP in chronic myeloid leukemia involves epigenetic modification and contributes to malignancy

**DOI:** 10.1038/cddis.2017.458

**Published:** 2017-10-12

**Authors:** Welbert O Pereira, Daniel D De Carvalho, Maria Emilia Zenteno, Beatriz F Ribeiro, Jacqueline F Jacysyn, Luiz R Sardinha, Maria A Zanichelli, Nelson Hamerschlak, Gareth E Jones, Katia B Pagnano, Fabiola A Castro, Yolanda Calle, Gustavo P Amarante-Mendes

**Affiliations:** 1 Departamento de Imunologia, Instituto de Ciências Biomédicas, Universidade de São Paulo, São Paulo, Brazil; 2 Instituto Israelita de Ensino e Pesquisa, Hospital Israelita Albert Einstein, São Paulo, Brazil; 3 Princess Margaret Cancer Centre, University Health Network, Toronto, Ontario, Canada; 4 Department of Medical Biophysics, University of Toronto, Toronto, Ontario, Canada; 5 Centro de Hematologia e Hemoterapia, Universidade de Campinas, Campinas, São Paulo, Brazil; 6 Faculdade de Medicina – LIM62, Universidade de São Paulo, São Paulo, Brazil; 7 Instituto de Tratamento do Câncer Infantil, Instituto da Criança, Hospital das Clínicas, Universidade de São Paulo, São Paulo, Brazil; 8 Departamento de Hematologia e Hemoterapia, Hospital Israelita Albert Einstein, São Paulo, Brazil; 9 Randall Division of Cell and Molecular Biophysics, King's College London, London, UK; 10 Faculdade de Ciências Farmacêuticas de Ribeirão Preto, Universidade de São Paulo, Ribeirão Preto, Brazil; 11 Department of Haemato-Oncology, King's College London, London, UK; 12 Instituto de Investigação em Imunologia, Instituto Nacional de Ciência e Tecnologia (INCT), São Paulo, Brazil

## Abstract

Chronic myeloid leukemia (CML) is a myeloproliferative disease caused by the BCR–ABL1 tyrosine kinase (TK). The development of TK inhibitors (TKIs) revolutionized the treatment of CML patients. However, TKIs are not effective to those at advanced phases when amplified BCR–ABL1 levels and increased genomic instability lead to secondary oncogenic modifications. Wiskott–Aldrich syndrome protein (WASP) is an important regulator of signaling transduction in hematopoietic cells and was shown to be an endogenous inhibitor of the c-ABL TK. Here, we show that the expression of *WASP* decreases with the progression of CML, inversely correlates with the expression of *BCR*–*ABL1* and is particularly low in blast crisis. Enforced expression of *BCR*–*ABL1* negatively regulates the expression of WASP. Decreased expression of WASP is partially due to DNA methylation of the proximal *WASP* promoter. Importantly, lower levels of *WASP* in CML advanced phase patients correlate with poorer overall survival (OS) and is associated with TKI response. Interestingly, enforced expression of *WASP* in BCR–ABL1-positive K562 cells increases the susceptibility to apoptosis induced by TRAIL or chemotherapeutic drugs and negatively modulates BCR–ABL1-induced tumorigenesis *in vitro* and *in vivo*. Taken together, our data reveal a novel molecular mechanism that operates in BCR–ABL1-induced tumorigenesis that can be used to develop new strategies to help TKI-resistant, CML patients in blast crisis (BC).

Chronic myeloid leukemia (CML) is a hematological malignancy caused by the chromosome translocation t(9:22) (q34;q11), which originates the oncogene *BCR*–*ABL1*. BCR–ABL1 is a protein composed of multiple domains involved in the initiation of a complex signaling transduction cascade.^1, 2^ The BCR portion of the oncoprotein contains a domain with serine and threonine kinase activity and regions that bind to Src homology-2 (SH2) domains, whereas the c-ABL portion contains SH3, SH2 and the tyrosine kinase (SH1) domains, in addition to four proline-rich domains, a nuclear translocation signal, a DNA- and an actin-binding domains.^3^ Binding of BCR–ABL1 to actin filaments was shown to contribute to transformation. Importantly, the tyrosine kinase (TK) activity is necessary for the transformation potential of BCR–ABL1^3, 4^ Particularly, BCR–ABL1 TK activity induces a strong resistance to both intrinsic and extrinsic pathways of apoptosis,^5^ for instance, by inducing overexpression of BCL-X_L_^6^ and MCL-1^7^ and downregulating TRAIL.^8^ Therefore, it is not surprising that the development of specific inhibitors directed to the TK catalytic site of BCR–ABL1, such as imatinib mesylate, revolutionized the treatment of CML patients.^9^

The evolution of CML comprehends three stages: chronic, accelerated and blast phases. In chronic phase (CP), the bulk of leukemic stem cells remains capable of undergoing differentiation, leading to the excessive production of mature granulocytes. In the accelerated phase (AP), differentiation becomes arrested, and the severe disease phenotype is caused by the proliferation of immature blasts, which increases in the BC.^3, 10^ Mechanisms of imatinib resistance are multifactorial and include point mutations within the BCR–ABL kinase domain,^11, 12^ BCR–ABL gene amplification,^11^ decreased intracellular drug availability^13^ and activation of alternative signaling oncogenic pathways.^14, 15^ In the advanced phases, patients present a stronger resistance to imatinib or other second- and third-generation TK inhibitors (TKIs),^16^ indicating an unmet need for improved therapeutic approaches in CML treatment.

BCR–ABL1 is the indispensable genetic modification responsible for CML,^3, 17, 18^ but other secondary^19^ genetic and epigenetic changes were also shown to contribute to malignancy, including resistance to TKIs.^20^ Although poorly understood, disturbance of the hematopoietic compartment is an evident phenomenon in CML that includes failure in differentiation of the stem cells in the bone marrow^21^ and inefficient function of mature leukocytes.^22, 23, 24^ Interestingly, these events are similar to those observed in patients with Wiskott–Aldrich syndrome (WAS), an immunodeficiency caused by the dysfunction of WAS protein (WASP).^25^ In addition, CML patients in BC also share symptoms with WAS patients, such as spontaneous bleeding, petechiae, ecchymosis, thrombocytopenia, enlargement of the spleen and increased susceptibility to infections.^26, 27, 28^ WASP is an adaptor protein exclusively expressed in hematopoietic cells capable of promoting actin polymerization.^29^ Important to our work, WASP was shown to act as an endogenous inhibitor of the TKs LCK, FYN and c-ABL.^30, 31, 32^

Here, we show that expression of WASP inversely correlates with BCR–ABL1 levels and the progression of the disease in CML patients. BCR–ABL1 downregulates *WASP* in part by epigenetic modification of its proximal promoter. Most importantly, downregulation of WASP contributes to the resistance to apoptosis and to BCR–ABL1-induced tumorigenesis. Therefore, WASP may serve as a molecular marker of prognosis, as well as a potential target for combined antitumor therapies for CML.

## Results

### WASP is downregulated in BCR–ABL1-positive cell lines and CML patients by a mechanism that involves epigenetic modification

**Figure 1:**
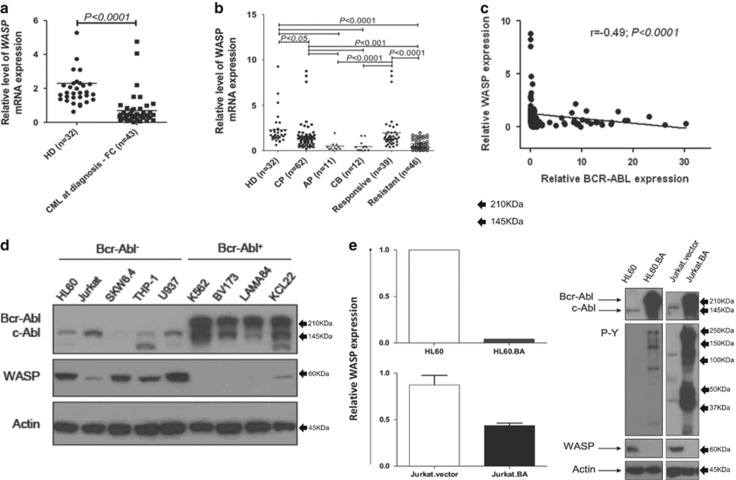
WASP is downregulated in CML patients and BCR–ABL1-positive cell lines. (**a**) The expression of *WASP* is suppressed in CML patients comparing with healthy donors (HD). The relative expression of WASP in PBMC was determined by real-time PCR using *GAPDH* as housekeeping gene (**P<0.01, comparing with HD). Values are plotted as 2^−ΔΔCt^. 't' student was used as statistical test. (**b**) The downregulation of WASP was observed in advanced phases of the disease (AP and BC). (*P*<0.0001, comparing with HD). Patients in CCyR showed normal WASP levels, according to the healthy donor control group (*P*<0.001, comparing with BP). Analysis of variance (ANOVA) and Bonferronni posttest were used to statistical analysis. (**c**) The expression of *WASP* negatively correlates with *BCR*–*ABL1* expression in CML patients (*P*<0.0001). Spearman test was used with linear regression. (**d**) Western blot showing the expression of WASP in cell lines derived from different leukemia types. BCR–ABL1-negative cell lines express high levels of WASP, whereas BCR–ABL1-positive cells express discrete or undetectable WASP. Actin was used as loading control. (**e**) Stable expression of *BCR*–*ABL1* was induced in HL-60 and Jurkat cell lines by retroviral infection, resulting in downregulation of WASP both at the mRNA and protein levels. *GAPDH* was used as housekeeping control for qPCR. Western blots show the increased numbers of tyrosine phosphorylated proteins in BCR–ABL-positive HL-60 and Jurkat cell lines, and the complete WASP silencing after *BCR*–*ABL1* expression. Actin protein was used as loading control

Initially, we sought to investigate the expression of *WASP* in CML patients and in BCR–ABL1-positive cell lines. We found that PBMC from CML patients expressed significantly lower levels of *WASP*, comparing with healthy donors (Figure 1a). CML patients in the CP presented lower levels of *WASP* and its expression was decreased during the progression of the disease to accelerated and blast phases (Figure 1b). Importantly, PBMC from patients resistant to TKI exhibited significantly lower levels of WASP compare with patients responsive to TKI (patients who achieved the complete cytogenetic remission (CCyR) and major molecular remission (MMR) after treatment with imatinib and dasatinib). Patients responsive to TKI were no different from either healthy individuals or CML patients at diagnosis (Figure 1b). These data indicate that *WASP* expression is linked to CML patient’s response to TKIs therapy and to *BCR*–*ABL1* levels. Moreover, we detected that patients who presented secondary resistance to TKI therapy (patients who achieved but subsequently lose relevant response) have higher *WASP* expression in comparison with patients who showed primary resistance (patients who did not reach a benchmark response) (Supplementary Figure 1). Twenty-one CML patients resistant to TKIs were tested for *BCR–ABL* mutation; 9 of them were positive for Y253H (2); M244V (2); T315I (2); F317L (1); H396R (1) e G250E/Y253H (1) mutations. There was no association with mutation *status* and *WASP* expression levels (*P*=0.97; Supplementary Figure 2).

In addition, the expression levels of *WASP* and *BCR*–*ABL1* were inversely correlated (Figure 1c), suggesting that *BCR*–*ABL1* could potentially be responsible for *WASP* downregulation. In order to investigate this possible cause–effect relationship, we evaluated WASP protein expression in cell lines derived from BCR–ABL1-negative leukemia (HL-60, Jurkat, SKW6.4, THP-1 and U937) and BCR–ABL1-positive CML patients (K562, BV173, LAMA-84 and KCL22). Unlike the BCR–ABL1-negative cells, WASP was strongly downregulated in BCR–ABL1-positive cell lines (Figure 1d) further supporting that BCR–ABL1 is a negative regulator of *WASP*. Our hypothesis was confirmed by transducing *BCR*–*ABL1* in the HL-60 and Jurkat cell lines, thereby producing HL-60.BCR–ABL1 and Jurkat.BCR–ABL1 cells. Enforced expression of *BCR*–*ABL1* induced a strong suppression of *WASP*, observed at both mRNA and protein levels (Figure 1e). Efficacy of *BCR*–*ABL1* transduction was confirmed by immunoblot using primary antibodies against c-ABL/BCR–ABL1 (to verify its expression) or to phosphotyrosine (to verify its activity) (Figure 1e). These results show that expression of *BCR*–*ABL1* inhibits *WASP* at both mRNA and protein levels.

**Figure 2:**
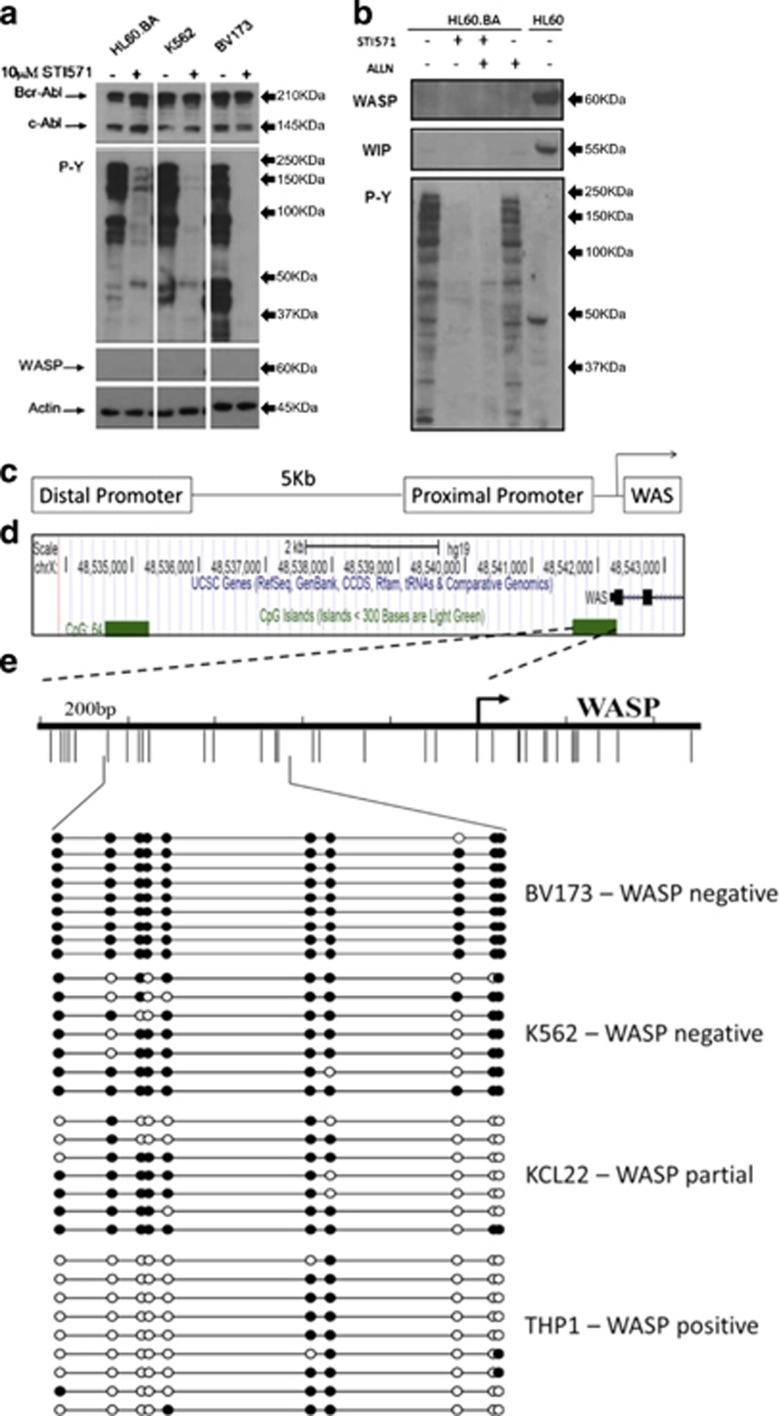
WASP downregulation is not rescued by imatinib or calpain inhibitor and involves epigenetic modification. (**a** and **b**) Cell lines were treated for 24 h with 10 *μ*M IM and/or the calpain inhibitor *N-*acetyl-l-leucyl-l-leucyl-l-norleucinal (ALLN), and western blot was performed to reveal the expression of WASP, WIP and the status of BCR–ABL1-TK activity. Actin was used as loading control. (**c**) Scheme of the distal and proximal promoters of WASP. (**d**) *In silico* analysis using UCSC Genome Browser public data (ww.ucsc.edu) show the presence of CpG sites in the promoters of WASP (green boxes). (**e**) CpG methylation status in the proximal promoter of WASP was evaluated. Black and white circles: methylated and unmethylated CpG sites

As most of BCR–ABL1-mediated signal transduction is dependent on its TK activity, we tested whether treatment with imatinib could reinstate the expression of *WASP* in BCR–ABL1-positive cells. To our surprise, the inhibition of the TK activity of BCR–ABL1 did not result in WASP re-expression in K562, BV173 or HL-60.BCR–ABL1 cells (Figure 2a), even in the presence of the dual calpain/proteasome inhibitor *N-*acetyl-l-leucyl-l-leucyl-l-norleucinal (Figure 2b), as protein levels of WASP is also tightly controlled by degradation via calpain-mediated proteolysis.^33^ These results suggest the emergence of an additional mechanism that maintains WASP inhibited independently of constant BCR–ABL1 TK activity.

DNA methylation at CpG sites localized in the promoter region is a well-known mechanism to stably suppress gene expression.^34^ As hematopoietic-specific expression of the *WASP* gene was shown to be driven by a promoter located upstream to the TSS^35^ (Figures 2c and d), we measured the DNA methylation levels at CpGs dinucleotide in a 400 bp CpG island positioned at this promoter region by bisulfite sequencing. We found an inverse correlation between DNA methylation of *WASP* promoter and gene expression. CML cell lines with suppressed *WASP*, such as BV173 and K562, presented the higher levels of DNA methylation: 99% and 77%, respectively. KCL22 (which express low levels of *WASP*) presented intermediated levels of DNA methylation: 54% (Figure 2e). In contrast, THP-1, a BCR–ABL1-negative cell line, which expresses high amounts of *WASP*, presented low level of DNA methylation: 22% (Figure 2e).

**Figure 3:**
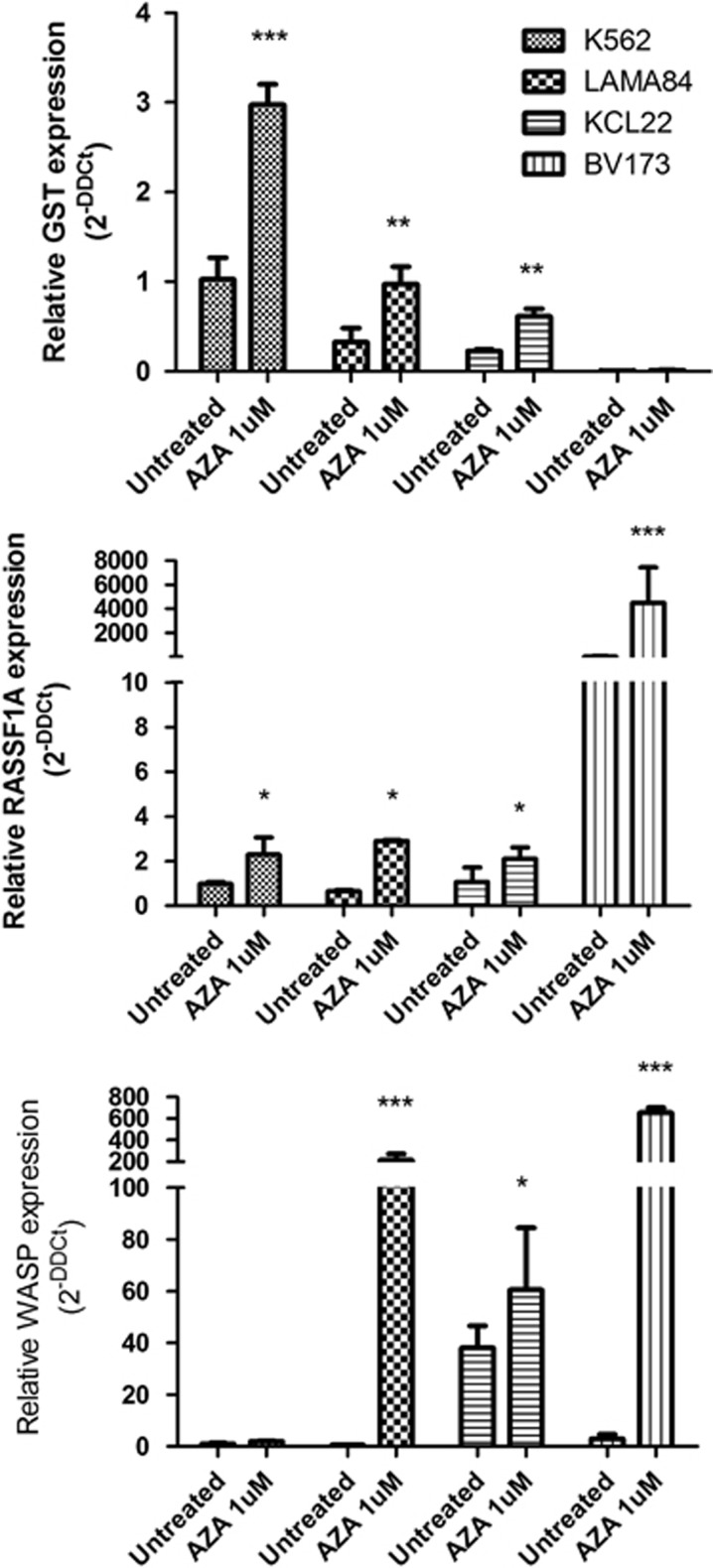
5-AZA treatment restored the levels of WASP expression in some but not all CML cell lines. (**a**) Treatment of CML cell lines with 1 *μ*M 5-AZA alone restored the levels of WASP expression in LAMA-84, KCL22 and BV173, but not in K562 cells. The relative expression of RASSF1A and GST was used as positive controls for 5-AZA treatment. Two way Anova (Analysis of variance) and Bonferroni post test were used to determine statistic significance among the groups. *P < 0.05, **P < 0.01, ***P < 0.001

Azacytidine (5-AZA) has presented significant therapeutic results for patients with acute myeloid leukemia and myelodysplastic syndromes, by inducing the demethylation of CpG sites and the consequent upregulation of silenced genes.^36, 37^ Treatment of CML cell lines with 1*μ*M 5-AZA alone restored the levels of *WASP* expression in LAMA-84, BV173 (greatly) and KCL22 (partially) cell lines, confirming that CpG methylation is indeed one epigenetic mechanism involved in BCR–ABL1-induced *WASP* suppression (Figure 3). Interestingly enough, 5-AZA failed to restore *WASP* levels in K562 cells, although it restored RASSF1A and GST control genes, suggesting the existence of a further mechanism responsible for downregulation of WASP in this cell line. Taken together, our data suggest that BCR–ABL1 inhibits the expression of *WASP* via multiple molecular mechanisms.

### Lower *WASP* expression in PBMC from CML patients in advanced phases correlates with poor OS and re-expression of *WASP* negatively modulates BCR–ABL1-induced tumorigenesis *in vitro* and *in vivo*

**Figure 4:**
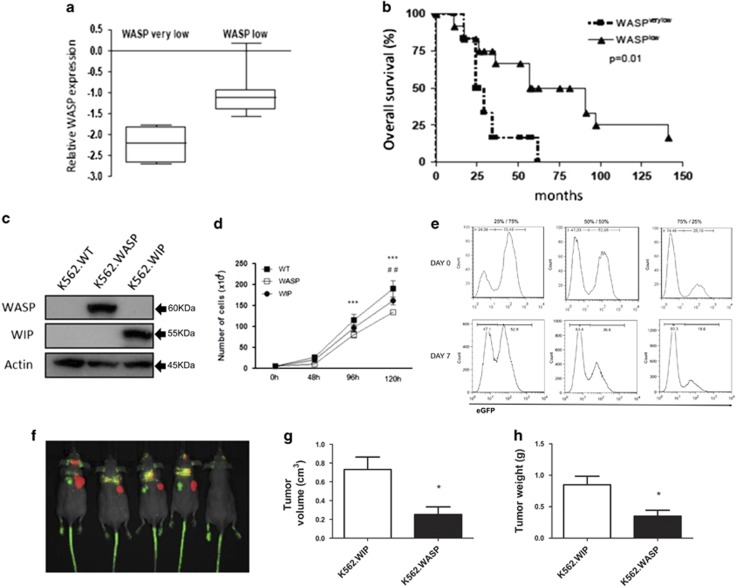
Lower levels of WASP in CML patients at advanced phases may indicate a worse OS and WASP re-expression negatively modulates BCR–ABL1-induced tumorigenesis. (**a**) The relative WASP expression values obtained from patients in AP and BP were divided in two groups according to the median: *WASP very low* for patients exhibiting a strong WASP downregulation (lower than median), and *WASP low* for patients presenting a mild WASP suppression (higher than median). (**b**) Patients with higher levels of WASP (grouped in *WASP low)* presented longer OS (median=61.75 months) comparing with the patients in *WASP very low* group. Kaplan–Meier was used as statistical test. (**c**) K562 cell line was transduced with lentivirus vector to induce the re-expression of WASP (eGFP) or WIP (mCherry). (**d**) K562.WT or re-expressing WASP or WIP were plated and the number of cells was counted in Neubauer chamber according to the time point in the graph. ***P<0.001 comparing K562.WASP with K562.WT. ^##^*P*<0.01 comparing K562.WASP with K562.WIP. (**e**) K562.WT was co-cultured with K562.WASP in three different initial proportions and the frequency of each population was assessed 7 days later by flow cytometry. (**f**) K562.WIP (mCherry-positive) and K562.WASP (eGFP-positive) were subcutaneously injected in the right and left flanks of the BALB/c nude mice, respectively, and the development of the solid tumors was followed and imaging of the tumors was assessed at 21^st^ day. The tumors were collected to determine volume (**g**) and weight (**h**). Student's *t-*test was used for statistical analysis. *P<0.05

Next, we investigated the biological relevance of decreased levels of *WASP* for CML patients. We analyzed the relationship between the levels of *WASP* with the OS of the 31 newly diagnosed CML patients and in 23 CML patients at AP and BC. For this analysis, we divided the patients in two groups according to the median of *WASP* expression. Patients with *WASP* levels below the median were called *WASP BM*, and patients with *WASP* levels above the median, were called *WASP AM*. There was no significant difference in OS according to WASP expression at CML diagnosis (Supplementary Figure 3A). In addition, we observed a tendency of a lower expression of *WASP* in high-risk Sokal patients (Supplementary Figure 3B). Interestingly, when the OS was calculated just for CML patients in advanced phases, resistant to TKIs and with significantly lower levels of WASP compared with healthy donors, we observed that milder suppression of *WASP* (levels above median – WASP low) correlated with longer OS, whereas strong suppression of this gene (levels below median – WASP very low) correlated with poorer OS (Figure 4b). Altogether, these data suggest that the downregulation of *WASP* by *BCR*–*ABL1* may be relevant to the CML prognosis, particularly for patients in advanced phases of disease.

Therefore, we investigated the consequences of re-expression of *WASP* on *in vitro* and *in vivo* models of BCR–ABL1-induced tumorigenesis. Using lentiviral transduction, we enforced the expression of eGFP-WASP or mCherry-WIP (as a control) in K562 cells, which lacks both WASP and WIP (WASP-interacting protein) (Figure 4c). First of all, the expression of WIP, a chaperone for WASP that prevents its targeting by calpain, thus increasing WASP half-life,^33^ did not result in increased expression of WASP in K562 cells, as expected after our results showing WASP promoter is silenced by DNA methylation in these cells (Figure 2e), as described above. Growth of tumor cells comprehends one important aspect of tumorigenesis and it is directly associated to the balance of proliferation and cell death *in vitro* and *in vivo*. In line with this, enforced expression of WASP decreased the growth of K562 cells compared with K562.WT and K562.WIP (Figure 4d). We also performed a mixed culture assay with 25%/75%, 50%/50% and 75%/25% of K562.WT (eGFP-negative) and K562.WASP (eGFP-positive), respectively. After 7 days of co-culture, the frequency of the cell populations was determined by flow cytometry. Under all the conditions tested, the relative percentage of K562.WT to K562.WASP increased with respect to the initial input (Figure 4e). In combination, these results suggest that re-expression of *WASP* decreases the robustness of the BCR–ABL1-positive cells, impacting its ability to grow *in vitro* (Figures 4c–e).

We next tested the impact of the re-expression of *WASP* in K562 cell growth *in vivo*, using a xenogeneic murine model of tumor growth. K562.WASP (eGFP-positive) and K562.WIP (mCherry-positive) were subcutaneously injected in the left and right flanks of BALB/c nude mice, respectively, and the growth of the tumors was followed up to 21 days. In agreement with the *in vitro* data, the expression of *WASP* in K562 drastically inhibited the *in vivo* tumor development, as assessed by *in vivo* imaging of the whole animal (Figure 4f), and by measuring the volume and weight of extracted tumors (Figures 4g and h).

Taken together, our data demonstrate that the expression of *WASP* has a negative impact on BCR–ABL1-induced tumorigenesis both *in vitro* and *in vivo*.

### *WASP* sensitizes BCR–ABL1-positive cells to TRAIL- and chemotherapy-induced apoptosis and interferes with BCR–ABL1 tumorigenic activity

**Figure 5:**
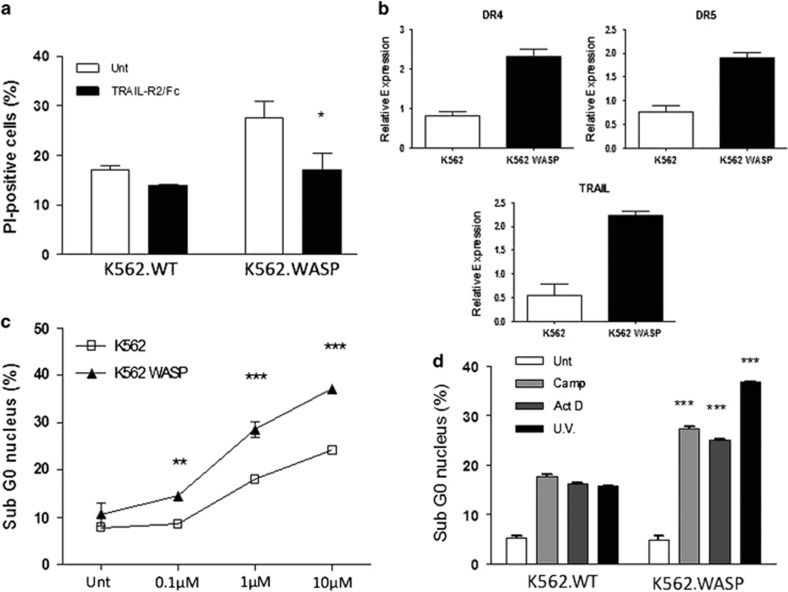
WASP sensitizes BCR–ABL1-positive cells to TRAIL- and chemotherapy-induced apoptosis. (**a**) K562.WT and K562.WASP were cultured in the presence or absence of TRAIL-R2/Fc to prevent TRAIL-mediated apoptosis and PI uptake was assesed 48 h later. (**b**) qPCR shows the upregulation of DR4, DR5 and TRAIL after re-expression of WASP in K562 cells. (**c**) K562.WT and K562.WASP were treated with different concentrations of IM and cell death was assessed by the hypodiploid DNA content (Sub G0) (*P<0.05; **P<0.01; ***P<0.001). (**d**) Cell lines were treated with camptothecin, actinomycin D and ultra violet (UV) radiation and cell death was assessed after 48 h by hypodiploid DNA content. Two way ANOVA was used for statistical analysis. ***P<0.001

To complete our study, we investigated the possible mechanisms involved in the negative impact of WASP on BCR–ABL1-mediated tumorigenesis. In fact, we observed a consistently increased 'spontaneous' apoptosis in cultures of K562.WASP cells compared with K562.WT (Figure 5a). This could be related to the fact that expression of WASP in K562 cells resulted in upregulation of the death receptors DR4 and DR5, and also their ligand TRAIL (Figure 5b). In fact, when we culture both cell lines in the presence of the chimeric TRAIL inhibitor, TRAIL-R2/Fc, we observed a reduction of PI-positive cells only in K562.WASP cell samples, and to the level found in K562.WT cultures (Figure 5a).

As TRAIL was shown to sensitize BCR–ABL1-positive cells to imatinib mesylate,^38^ we tested whether the expression of WASP in K562 cells rendered them more sensitive to imatinib. Indeed, imatinib treatment at a dose range achievable in patients resulted in higher frequency of apoptotic K562.WASP cells compared with K562.WT (Figure 5c). Finally, we tested whether the expression of WASP could also sensitize K562 cells to chemotherapy-induced apoptosis. Similarly, K562.WASP cells were more susceptible to apoptosis induced by camptothecin, actinomycin D and UV irradiation than K562.WT cells (Figure 5d). Therefore, increased levels of apoptosis appear to account for the tumor-suppressor activity mediated by WASP. These results indicate that the expression of WASP sensitizes BCR–ABL1-positive cells to apoptosis.

## Discussion

The development of TKI was a turning point for the treatment of CML. However, its efficiency depends on the early diagnosis, early administration and continuing treatment. In addition, part of the CML patients do not respond to imatinib or second- or third-generation TKIs.^16^ Therefore, the discovery of novel signaling pathways associated with BCR–ABL1-induced tumorigenesis is still a matter of importance for patients with BCR–ABL1-positive leukemia. We show here for the first time that WASP may serve as a molecular marker of prognosis, as well as a potential target for combined antitumor therapies for CML.

Initially, we demonstrated that BCR–ABL1 inhibits the expression of WASP both at mRNA and protein levels. Particularly important is the fact that downregulation of WASP seems to take place mostly at the transition of chronic to advanced phases of the disease. At this point, increased genomic instability may result in additional genetic abnormalities that are discretely acquired with no significant changes in the clinical aspects of disease. This includes no relevant increase in blast numbers, making it difficult to recognize the molecular evolution of CML patients.^16, 39, 40^ The observed inhibition of WASP expression coincided exactly with this transition, as patients in CP still express WASP at levels similar to healthy individuals, whereas patients in AP and BC has significantly less WASP (Figure 1b). Therefore, monitoring the expression of WASP could be useful as a molecular marker of progression of CML and may help in future therapeutic decisions, such as early switch to more potent TKI or to use combining chemotherapies.

Besides its potential use as a biomarker for AP/BC, WASP suppression seems to have a role in the severity of the disease. First, as mentioned above, WASP levels become significantly lower in advanced phases of CML (AP+BC). Most importantly, by dividing these two groups of patients according to their (diminished) expression of WASP, we observed that those with very low levels of WASP (below the median) had a significantly shorter OS than those with low levels (above the median), independently of the stage of the disease (Figures 4a and b). Interestingly, another member of the WASP family, N-WASP, has been implicated in the progression of breast and colorectal cancer.^41, 42, 43^ Similar to our results, the authors showed a strong correlation between low levels of N-WASP and shorter OS/disease-free survival. However, they linked the increased severity of the disease to an augmented effect of the absence of N-WASP on tumor invasiveness/metastasis, which clearly does not apply to our case, because of the leukemic nature of CML.

The enforced expression of WASP in K562 cells revealed the biological consequences of WASP suppression in CML. Our data demonstrate that the strong resistance to apoptosis conferred by BCR–ABL1 to leukemic cells was attenuated by the introduction of a WASP-expressing construct. Besides inducing TRAIL-dependent spontaneous apoptosis, with participation of the increased expression of DR4 and DR5, recovery of WASP turned K562 cells sensitive to different chemotherapeutic drugs, as well as to imatinib. These data suggest that re-activation of WASP could be an interesting therapeutic strategy in CML, especially in combination with imatinib or other TKI. Interestingly, the deficiency of WASP expression has already been reported to induce or to facilitate apoptosis in other contexts.^44, 45, 46^

In order to explore this possibility, we investigated the mechanisms behind WASP downregulation in CML. To our surprise, treatment of CML cell lines with imatinib did not show any sign of recovery of WASP, suggesting that the inhibition of WASP by BCR–ABL1 is either a kinase-independent event or may undergo a secondary layer of regulation that does not depend on the constant kinase activity of BCR–ABL1. The existence of kinase-independent mechanisms derived from the expression of BCR–ABL1 has been described.^47^ For instance, BCR–ABL1 can bind and activate HCK independently of its catalytic activity.^48^ Moreover, the expression of BCR–ABL1, but not its kinase activity, was shown to be important to the survival of quiescent CML stem cells.^49^ In line with this, we have shown that robust pharmacological inhibition of BCR–ABL1 TK, as assessed by the disappearance of phosphotyrosine-containing proteins, does not interfere with the strong resistance to apoptosis observed in BCR–ABL1-positive cell lines, at least for 24 h, when levels of BCL-X_L_ were significantly reduced.^50, 51^

The stability of WASP suppression in all tested CML cell lines led us to investigate the existence of an epigenetic inhibitory mechanism. Indeed, we found that a CpG island located at WASP promoter was strongly methylated in BV173 and K562 cell lines, implicating DNA methylation as a novel mechanism for WASP inhibition.

Re-expression of WASP in the BCR–ABL1-positive cells hampers both the *in vitro* and *in vivo* tumorigenic potential, and increases sensitivity to imatinib and to chemotherapeutic drugs, providing a rationale for the development of new therapeutic strategies aiming to restore the levels of WASP in BCR–ABL1 leukemia. Our data suggest that one interesting strategy is combining Azacytidine, an FDA-approved DNA demethylating agent, to increase WASP expression with TKIs, such as imatinib. Another potential strategy is using gene therapy approaches to correct WASP expression. Interesting enough, γ-retroviral and HIV-based lentiviral vectors designed to correct WASP levels are already in place for patients with WAS.^52, 53, 54^

One final consequence of our findings is related to proposed immunotherapy using dendritic cells isolated from CML patients.^55, 56, 57^ Although there are evidences that the immune system can potentially eliminate CML cells, particularly imatinib-resistant BCR–ABL1-positive stem cells, a note of caution should be added to the use of autologous dendritic cells, as patients in advanced phase of CML express low levels of WASP and, therefore, are likely to present impaired immunological synapses and consequent deficiency in antigen presentation. In fact, using a retrovirus-induced murine model of CML, Mumprecht and collaborators have shown that BCR–ABL-expressing DCs do not efficiently induce specific CTL immune response.^22, 23, 24^

Taken together, we described a new tumor-suppressor activity for WASP and demonstrated that its expression in CML interferes with BCR–ABL1-mediated signaling transduction, increases the sensitivity of leukemic cells to apoptosis and is associated with a better prognosis of CML patients. Therefore, the strategies aimed to recover the expression of WASP, which are currently being tested for WAS patients,^58^ may be potentially considered as a therapeutic approach for some CML patients.

## Materials and Methods

### Patients and controls

WASP expression was analyzed in 32 healthy individual and 85 CML patients (62 CP, 11 AP and 12 crisis blastic phase) treated at the Euryclides de Jesus Zerbini Transplantation Hospital and the Hematology and Hemotherapy Center, University of Campinas, State of São Paulo, Brazil, between April 2003 and October 2010. One patient was diagnosed in August 2015. Thirty-nine patients were responsive to TKIs therapy (imatinib or dasatinib) and 46 were resistant. Most of patients from responsive groups presented a major molecular response after dasatinib therapy. The demographic and clinical features of evaluated patients are shown at Tables 1 and 2 (Supplementary Data).

The study was approved by the local research ethics committees and patients gave written informed consent for their participation, in accordance with the Declaration of Helsinki. Demographic and disease characteristics were collected at baseline. CML diagnosis of the patients enrolled in this study was confirmed by the demonstration of Philadelphia chromosome in conventional cytogenetics and/or *BCR–ABL1* detection by RT-PCR. Hematologic, cytogenetic and molecular responses were redefined according to the European LeukemiaNet 2013 recommendations.

Peripheral blood mononuclear cells from patients and controls were isolated according to standard protocol with the Ficoll–Hypaque 1077 density technique.

### Cell lines

All cell lines K562, LAMA-84, KCL22, BV173, Jurkat, HL-60, SKW6.4, THP-1 and U937 were cultured in RPMI medium 1640 supplemented with 10% fetal bovine serum, 25 mM Hepes, 2 mM l-glutamine, 100 U/ml penicillin and 100 *μ*g/ml streptomycin. HL-60 and K562 cells were obtained from ATCC (Manassas, VA, USA). LAMA-84 was kindly provided by Dr. James Griffin (Dana-Farber Cancer Institute, Boston, MA, USA). HL-60.BCR–ABL1 cells were derived from wild-type HL-60 by retroviral transfection with pSRαMSVp185^BCR^^–ABL1^tkneo, as previously described.^51, 52^ K562 were infected with lentivirus vectors to induce expression of WASP.GFP and WIP.mchery,^59^ and sorted using FACS ARIA (Becton-Dickinson, Franklin Lakes, NJ, USA) to obtain high purity cell lines.

### Reagents

For western blot, we used anti-WASP (Santa Cruz Biotechnologies, Dallas, TX, USA), anti-WIP (Santa Cruz Biotechnologies), anti-c-ABL OP-20 (Oncogene Research Products, La Jolla, CA, USA), anti-Actin (Sigma Aldrich, St. Louis, MO, USA). TRAIL-R2/Fc protein was generously provided by Dr. Henning Walczak (Imperial College, London, UK).

### Quantitative PCR

Total RNA was extracted using Trizol (Invitrogen, Carlsbad, CA, USA). RNA concentration and purity were determined spectrophotometrically by measuring fluorescence at 260 and 280 nm. Three micrograms of RNA was reverse transcribed into cDNA using Superscript III (Invitrogen) transcription reagents according to the manufacturer’s instructions. After obtaining the cDNA, gene expression was quantified by quantitative PCR using Platinum SYBRGreen Kit (Invitrogen) in Mx3005P detector equipment (Stratagene, Santa Clara, CA, USA). The following primers were used: BCR–ABL 5′-TGGGTCCAGCGAGAAGGTT-3′ (forward) and 5′-GCATTCCGCTGACCATCAAT-3′ (reverse); GAPDH 5′-GGAGAAGGCTGGGGCTCAT-3′ (forward) and 5′-TCCTTCCACGATACCAAAGTT-3′ (reverse); TRAIL 5′-AAGGCTCTGGGCCGCAAAATAAAC-3′ (forward) and 5′-CCAACTAAAAAGGCCCCGAAAAA-3′ (reverse); DR4 5′-GTACGCCCTGGAGTGACATC-3′ (forward) and 5′-CCTCGTAGGAGACCCAAGC-3′ (reverse); DR5 5′-CTAGCTCCCCAGCAGAGAGT-3′ (forward) and 5′-GTGGTGCAGGGACTTAGCTC-3′ (reverse). WASP 5′-GGCTGGTCGGCTGCTCTGGGAACA-3′ (forward) and 5′-GGTGGTGGGGGTAGCTGGCGTCTGT-3′ (reverse). Results were given as relative expression represented as 2^−ΔΔCt^.

### Western blot

Protein samples were resolved under reducing conditions as previously described.^45^ Separated proteins were transferred onto polyvinylidene difluoride membranes and reactions were detected with a suitable secondary antibody conjugated to horseradish peroxidase (Jackson Laboratory, Bar Harbor, ME, USA and Amersham, Arlington, IL, USA) using enhanced chemiluminescence (Pierce, Rockford, IL, USA).

### Treatments

Cell lines were treated with imatinib mesylate (0.1, 1 and 10 *μ*M), actinomycin D (5 *μ*M), camptothecyn (100 *μ*M) and UV.C. (400 J/cm^2^) for 48 h and cell death was determined by flow cytometry. For TRAIL pathway blockage, cell lines were treated with 5 *μ*g/ml of TRAIL-R2/Fc for 48 h.

### Assessment of cell death

Apoptosis was quantified using the fluorescence-activated cell sorter (FACS) Calibur flow cytometer (Becton-Dickinson), by analysis of DNA content as described.^51^ We also evaluated cell death by propidium iodide (PI) incorporation for dead cells using flow cytometry. The results represent the average±S.D. in triplicate samples. Every experiment was repeated at least three times.

### Cell growth analysis and proliferation assays

For absolute count of cells, 4 × 10^4^ cells K562.WT, K562.WASP, K562.WIP and K562.WASP.WIP were cultured in six-well plates with 1 ml of medium, and after each 24 h, 0.5 ml of fresh medium was added to avoid starvation and cellular stress. The absolute number of the cells was counted in Neubauer chamber each 24 h during 5 consecutive days. For the co-culture assays, 2 × 10^5^ cells K562.WT (GFP negative) and K562.WASP (GFP positive) were co-cultured in three different proportions: 25%:75% 50%:50% 75%:25%, respectively. The cells were homogenized by pipetting, half part was discarded and the culture received fresh medium every 24 h. After 7 days of assay, the frequency of the populations was evaluated by flow cytometry and compared with day 0. For proliferation assay, K562.WT and K562.WASP were stained with *Violet Cell Trace* (Molecular Probes, Invitrogen, Carlsbad, CA, USA) and cultured for 4 days, and the proliferation rate was determined by fluorescence decay using flow cytometry analysis. The results represent the average±S.D. in triplicate samples. Every experiment was repeated three times.

### Animals and *in vivo* model of K562-induced solid tumors

Five million K562.WASP.gfp and K562.WIP.mcherry cells were subcutaneously injected in the left and right superior flanks of 6–8-week-old, female BALB/c nude mice, respectively. The tumor growth was followed during 21 days, when the animals were killed and the tumors were removed to determine weight and volume. Images of the animals were collected at day 18 after inoculation, and tumors were analyzed through detection of gfp and mcherry fluorescence using IVIS Spectrum Pre-clinical *In Vivo* Imaging System (Perkin Elmer, Waltham, MA, USA). Groups of at least 6 mice were used in each experiment, which was repeated three times. All animals were housed at our animal facility at the Institute of Biomedical Sciences, University of Sao Paulo (ICB-USP). This study was carried out in strict accordance with the recommendations in the Guide for the Care and Use of Laboratory Animals of the Brazilian National Council of Animal Experimentation (*http://www.cobea.org.br/*). The protocols were approved by the Animal Ethics Committee of the ICB-USP.

### Methylation analysis in WASP promoter

One *μ*g of genomic DNA from each cell line was bisulfite converted using the Zymo EZ DNA methylation kit (Zymo Research, Orange, CA, USA), according to the manufacturer’s instructions. To analyze the DNA methylation status of individual DNA molecules, we amplified by PCR a 400 bp region upstream to WAS TSS after the bisulfite conversion. The PCR fragments were cloned into the pCR2.1 vector using the TOPO-TA cloning kit (Invitrogen). Individual colonies were screened for the insert and the region of interest was sequenced using M13 primers as previously described.^60^

### 5’Azacytidine treatment and induction of DNA demethylation

The cell lines K562, LAMA-84, KCL22 and BV173 were treated with 1 *μ*M of Azacytidine for 120 h. The supplemented medium was refreshed every 24 h in order to maintain the drug concentration during the experiment. Cells were harvested and RNA was obtained. Real-time PCR was performed to investigate the recovery of *WASP* after DNA demethylation. Glutatione *S*-tranferase-pi 1 (GSTP1) and Ras association domain family member 4 (RASSF4), which are described as suppressed genes by CpG methylation in CML cell lines^61, 62, 63^ were used as control of the efficacy of the treatment, and *GAPDH* as the housekeeping control.

### Statistical analysis

The probability of OS of 31 CML newly diagnosed patients (clinical and laboratorial features at Table 3,Supplementary Data) according to *WASP* expression levels was calculated from CML diagnosis until death or last follow-up using the Kaplan–Meier method and the log-rank test, using IBM SPSS software, version 21 (IBM, Armonk, NY, USA). We also calculated the OS of 23 CML patients in advanced phases (AP and BP) resistant to IM 400 mg daily according to WASP expression levels.

*WASP* gene expression analysis among healthy individuals and patients' different groups were performed by using the GraphPad Prism software version 7 (GraphPad Software Inc., La Jolla, CA, USA).

## Figures and Tables

**Table 1:** Demographic, clinical and laboratorial features of CML patients resistant to tyrosine kinase inhibitor therapy

		***n***	**%**
Age (median, years)	54 (21–77)		
Gender	Male	27	59
Phase	Chronic	23	50
	Accelerate	11	24
	Blast crisis	12	26
Resistance	Primary	37	80
	Secondary	9	20
Treatment	Imatinib (400 mg daily)	29 (3^a^)	63%
	Dasatinib (100–140 mg/day)^a^	8	17%
	Nilotinib (400 mg BID)^a^	3	11%
	Hydrea (1–2 g/day)^a^	5	7%
	Interferon (4.5 million/U/day)^a^	1	2%
Mutation		9/21^b^	43

a After previous failure with others TKIs

b Mutations: 2 Y253H; 2 M244V; 2 T315I; 1 F317L; 1 H396R e 1 G250E/Y253H

**Table 2:** Demographic, clinical and laboratorial features of CML patients responsive to tyrosine kinase inhibitors therapy

		***n***	**%**
Age (median, years)	43 (19–73)		
Gender	Male	24	62
Phase	Chronic	39	100
Response	CCyR	18	46
	MMR	21	54
Treatment	Imatinib (400 mg/daily)	18	46
	Dasatinib	21	54

Abbreviations: CCyR, complete cytogenetic remission; MMR, major molecular remission

**Table 3:** Clinical and laboratorial features of 31 newly diagnosed CML patients

		***n***	**%**
Age (median, years)	48 (21–69)	31	
Gender	Male	18	58
Phase	CP	30	96.8
	AP	1	3.2
Sokal score	Low	10	32.3
	Intermediate	8	25.8
	High	6	19.4
	Missing	7	22.6
First TKI treatment	Imatinib	26	83.9
	Bosutinib	4	12.9
	Nilotinib	1	3.2
WASP expression	Low	16	51.6
	High	15	48.4
WASP relative expression. (median, range)	0.328 (0.008–0.778)	31	
