# Supplementary figures and images for: BCR–ABL1-induced downregulation of WASP in chronic myeloid leukemia involves epigenetic modification and contributes to malignancy
Source: Cell Death Dis. 2017 Oct 12;8(10):e3114–. doi: 10.1038/cddis.2017.458 (PMC5680580; doi:10.1038/cddis.2017.458)

Supplementary Figure 1.

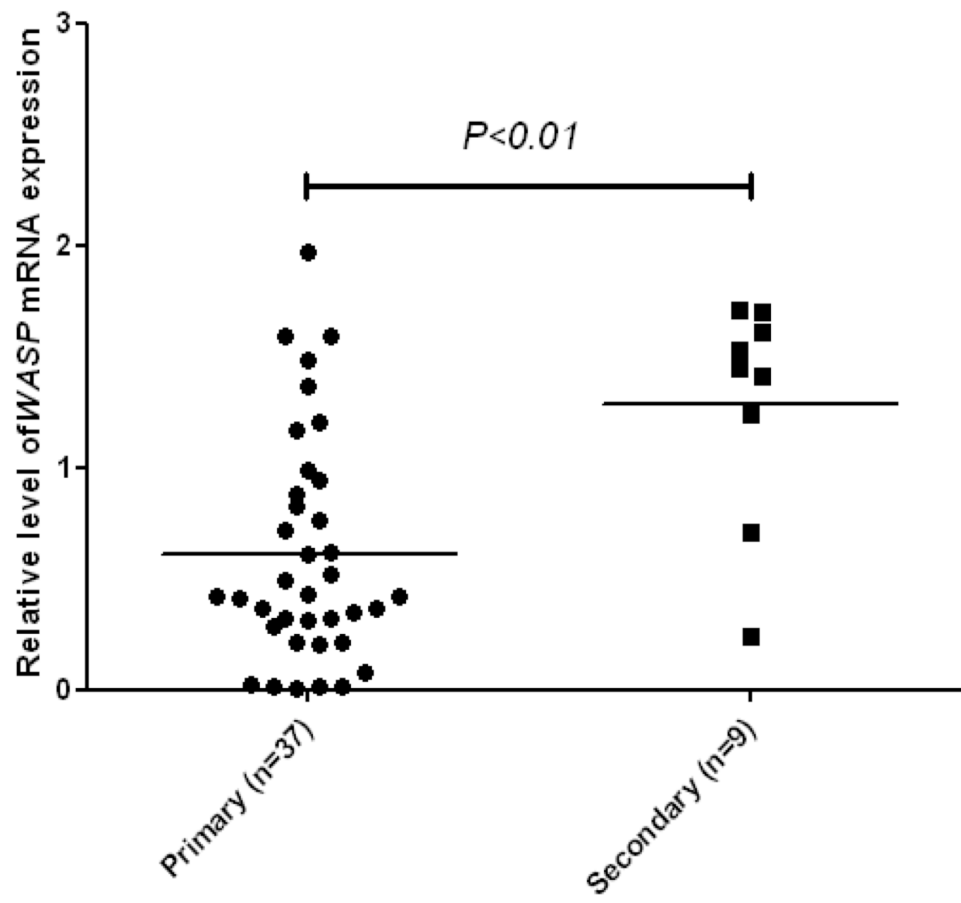

Supplement: Supplementary Figure 1 [file cddis2017458x1.pdf]

Supplementary Figure 2

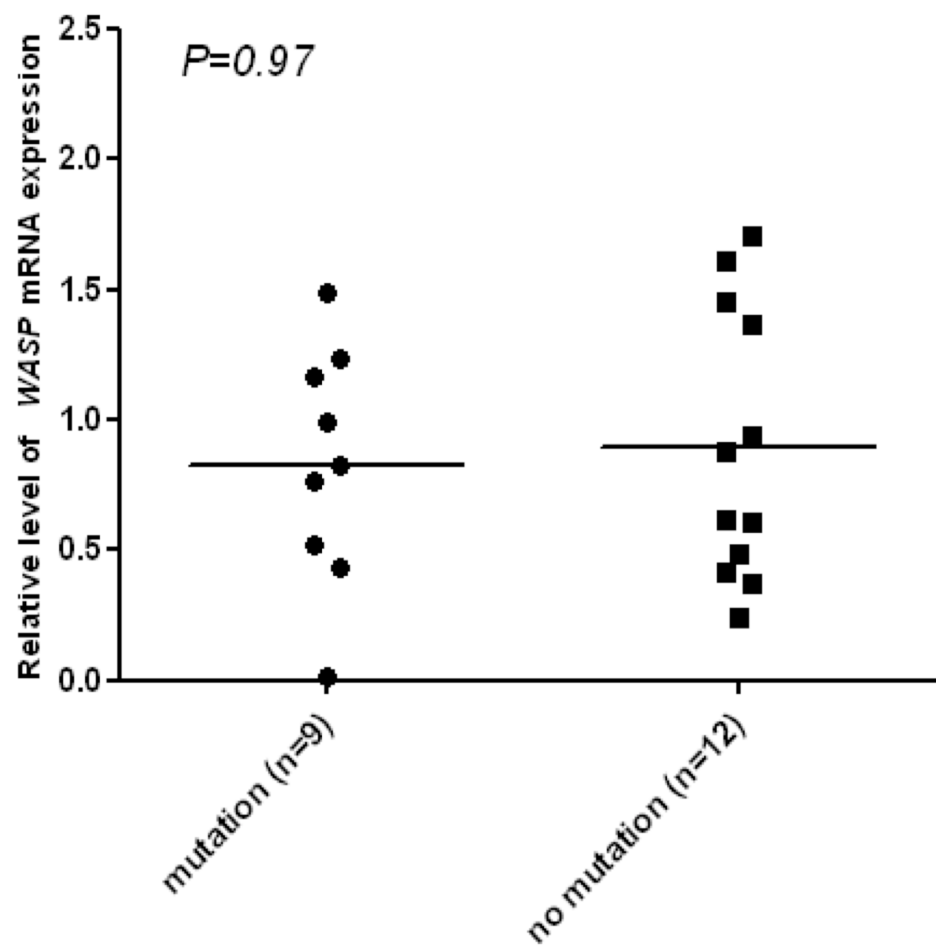

Supplement: Supplementary Figure 2 [file cddis2017458x2.pdf]

Supplementary Figure 3

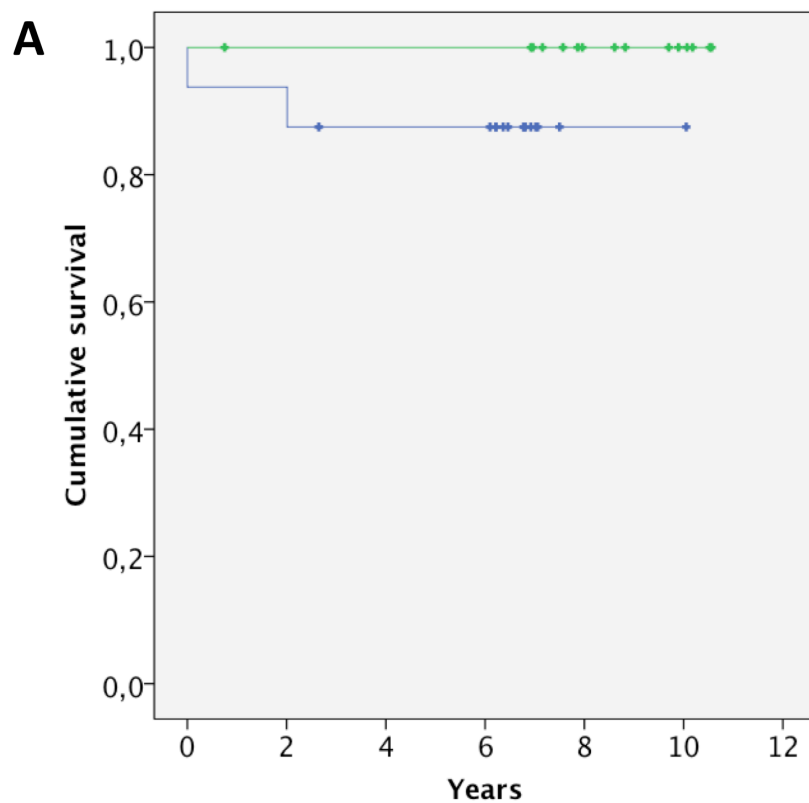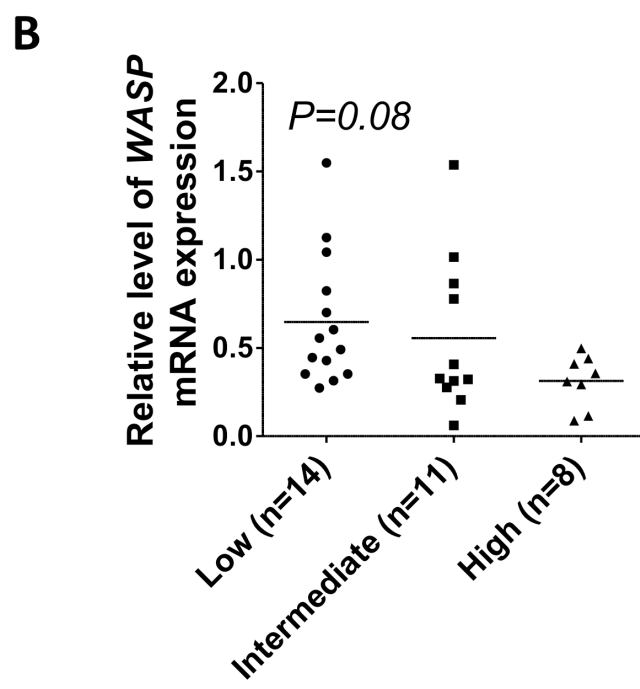

Supplement: Supplementary Figure 3 [file cddis2017458x3.pdf]
