# Supplementary material for: BCR–ABL1-induced downregulation of WASP in chronic myeloid leukemia involves epigenetic modification and contributes to malignancy
Source: Cell Death Dis. 2017 Oct 12;8(10):e3114–. doi: 10.1038/cddis.2017.458 (PMC5680580; doi:10.1038/cddis.2017.458)
Supplement: Supplementary Figure Legends [file cddis2017458x4.docx]

**Supplementary Figure 1: WASP is up regulated in CML patients with secondary resistance to imatinib mesylate.** The *WASP* expression is higher in patients with secondary resistance to imatinib mesylate (patients who achieved but subsequently lose relevant response, such as loss of cytogenetic or hematologic response and progression from chronic to advanced-stage disease) in comparison with patients with primary resistance to IM (patients who fail to achieve a landmark response, such as hematologic response and major or complete cytogenetic response in early chronic phase CML treated with imatinib at diagnosis) (p<0.01). The relative expression of *WASP* in PBMC was determined by Real Time PCR using *GAPDH* as housekeeping gene Values are plotted as 2^-ΔΔCt^. “t” student was used as statistical test.

**Supplementary Figure 2- *BCR-ABL* mutation *status* does not interfere in *WASP* expression.** There was no differential expression of *WASP* between CML patients harbouring *BCR-Abl* mutations and CML patients with no *BCR-ABL* mutation (p=0.97)*.* The relative expression of *WASP* in PBMC was determined by Real Time PCR using *GAPDH* as housekeeping gene Values are plotted as 2^-ΔΔCt^. “t” student was used as statistical test.

**Supplementary Figure 3 – (A) *Overall survival according to WASP expression at diagnosis (P=NS)*.** The relative *WASP* expression values obtained from 31 newly diagnosed CML patients were divided in two groups according to the median: *WASP BM* for patients exhibiting *WASP* levels below than median (blue line) and *WASP AM* for patients presenting a *WASP* expression above median (green line). Patients’ probability of overall survival (OS) was calculated for the two groups from diagnosis until death or last follow-up using the Kaplan-Meier method and the log-rank test, using IBM SPSS software, version 21. There was no significant difference in OS according to *WASP* expression at diagnosis (p>0.05). **(B) *WASP expression according to Sokal risk group at diagnosis*.** The *WASP* expression was evaluated in the 33 untreated CML patients according SOKAL score: low, intermediate and high (p=0.08). There was a tendency of a lower expression of *WASP* in high-risk Sokal patients. Anova test was used as statistical test.
